# Amylose-Lipid Complex as a Fat Replacement in the Preparation of Low-Fat White Pan Bread

**DOI:** 10.3390/foods9020194

**Published:** 2020-02-14

**Authors:** Hee-Seon Lee, Kyung-Heon Kim, Sung-Hoon Park, Sung-Won Hur, Joong-Hyuck Auh

**Affiliations:** 1 Department of Food Science and Technology, Chung-Ang University, Anseong 17546, Korea; angel2hs@naver.com (H.-S.L.); l313ove@nate.com (K.-H.K.); 2 SPC Research Institute of Food and Biotechnology, Seoul 08826, Korea; parksh@spc.co.kr (S.-H.P.); nutrissung@spc.co.kr (S.-W.H.)

**Keywords:** amylose-lipid complex, shortening, low calorie, bread

## Abstract

Amylose-lipid complex (ALC) was prepared with corn starch and stearic acid and used as a shortening replacement in white pan bread preparation. ALCs were prepared using various concentrations of stearic acid to corn starch (1%, 3%, 5%, and 7%) under different temperatures (55, 65, and 75 °C) and for different durations of time (30, 60, and 120 min); then, their complexing properties were assessed using iodine reagent and X-ray diffraction. The complexing reaction at 75 °C for 60 min showed the highest complexing index of the tested conditions; the in vitro digestibility of ALC was lower than that of corn starch. White pan bread was prepared with ALCs and their characteristics, including appearance, loaf volume, and starch retrogradation during storage at room temperature for four days, were compared with those of control bread. With increasing ALC replacement concentrations, loaf volume and shape were significantly affected; however, starch retrogradation was significantly retarded and energy value decreased by ALC replacement. Overall, 50% replacement of shortening by ALC appeared to be a reasonable level for retaining the basic characteristics of the bread while retarding the staling process. These results indicate that ALCs may be potentially useful in the bakery industry for preparing low calorie and low-fat products.

## 1. Introduction

Bread is the most important staple food worldwide and is recognized as a perishable food, best when consumed ‘fresh’. Bread is an unstable, elastic, solid foam. Its solid part contains a continuous phase comprising an elastic network of cross-linked gluten molecules and leached starch polymer molecules, primarily amylose, both uncomplexed and complexed with polar lipid molecules and a discontinuous phase of entrapped, gelatinized, swollen, and deformed (wheat) starch granules [1]. Shortening, an edible fat, is an important ingredient in bakery products such as cakes, cookies, and pastries [2]. It is also known as vegetable shortening and is made up of 100% fats from refined vegetable oils blended with two or more partially hydrogenated oils [3]. Shortening is one of the most important ingredients in producing bread. The liquid matter in shortening has a lubricating effect on bread, providing a moister mouthfeel during chewing. It tenderizes the crumb of cooled bread to provide a tender and well aerated bakery product by enabling the lubrication of gluten particles as it breaks the continuity of the protein and starch structure [4]. Further, it tenderizes the crust by counteracting the tendency of moisture to migrate from the center of the loaf to the drier surface region, which causes a leathery consistency during storage [3]. Shortening also increases volume by shortening the strands of gluten and foaming and whipping agents during the aeration process of dough preparation [5].

Recently, with increasing consumer demand for healthy foods, such as low-calorie and low-fat products, a great deal of effort has been made to reduce the use of shortening owing to its high level of saturated fatty acids as well as the possible presence of trans fatty acids [6]. High intake of trans fatty acids was found to be positively correlated with diabetes in age- and BMI-adjusted analyses [7]. Many studies have reported reductions in the use of shortening in bread making using various replacements. Oat β-glucan amylodextrins (oatrim) were used as a substitute for shortening in cakes and chocolate chip cookies [8,9], and canola oil-carnauba wax oleogels were evaluated as a replacement for shortening in a baked cake system [10]. Additionally, the carbohydrate-based fat replacer Z-Trim, the protein-based fat replacer microparticulated protein, and the lipid-based fat replacer salatrim have attracted attention as candidates for fat replacement in food [11].

As fat has a higher caloric density than most nutrients in foods [12], reducing the fat and cholesterol content is currently one of the primary trends in food product innovation [13]. Many studies have reported the replacement of shortening with various substitutes. Flaxseed oil, being the richest vegetarian source of alpha linolenic acid (omega-3 fatty acid), was incorporated into cookies to replace shortening [14]. Tanti, et al. [15] reported that cookie creams with desirable functional behavior could be made by replacing shortening with hydroxypropyl methylcellulose and methylcellulose-structured canola oil at intermediate levels.

However, to our knowledge, no attempt to use amylose-lipid complexes (ALCs) as a replacement for fats, especially shortening, in bread preparation has been reported. The formation of starch-lipid complexes is a well-known interaction, and complexation mainly occurs between amylose and lipids [16]. The characteristics of amylose-lipid complexes are typically influenced by the purity of the starting materials and the complexation reaction conditions [17]. Most studies on amylose-lipid complexes mainly focused on the preparation and characterization of these inclusion complexes. Previous reports on the formation of amylose-lipid complexes and their crystalline properties reported that they are significantly influenced by numerous factors, especially amylose chain length, fatty acid chain length and saturation, reaction time, pH, and incubation temperature [17,18,19]. In addition, specially designed complexes, such as the so-called V-amylose complexes, have shown potential in various food-related applications, such as the nanoencapsulation of sensitive bioactive or flavor compounds, the formation of amylose nanotubes, and the modification of starch rheological functionality [20].

Recently, ALC was recognized as a new type of resistant starch (resistant starch 5); a starch resistant to enzymatic digestion [21]. Resistant starch 5 (RS5) is reported to reduce postprandial glycemic responses and have potential as an intervention in metabolic syndromes, such as type 2 diabetes, obesity, hypertension, and heart disease [21]. Additionally, ALC significantly retarded starch retrogradation and staling of stored tortillas via formation of inclusion complexes between amylose and lipid [22].

In this study, ALC was prepared from corn starch and stearic acid under different conditions including stearic acid-starch ratio (1%, 3%, 5%, and 7%), temperatures (55, 65, and 75 °C), and duration time (30, 60, and 120 min) and its potential as a shortening replacement in bread making was investigated.

## 2. Materials and Methods

### 2.1. Materials and Reagents

Corn starch was purchased from Daesang Co., Inc., Seoul, Korea. Food-grade stearic acid was purchased from Sigma-Aldrich, St. Louis, MO, USA. All reagents were chemical-grade; iodine (99%) was obtained from Showa Chemical Industry Co., Ltd., Japan, and potassium iodine (99.5%) was purchased from Junsei Chemical Co., Ltd., Tokyo, Japan.

### 2.2. Preparation of the Amylose-Lipid Complex (ALC)

ALC was prepared as described in previous studies with some modification [19,23]. A 500 mL of 5% (*w*/*w*) starch slurry was prepared with corn starch and pre-warmed water (40 °C); then, a gelatinized starch-water mixture was prepared by stirring for 30 min at approximately 95 °C using an overhead stirrer (Kipae E&T Co., Ltd., Hwaseong, Korea). Gelatinized starch slurries were cooled to different temperatures (55, 65, and 75 °C), and a stearic acid solution (25 mL of 5%) was prepared with ethyl alcohol. The stearic acid solution was added to the gelatinized starch slurries, which were then continuously stirred for 30, 60, and 120 min at each temperatures (55, 65, and 75 °C). After the reaction, the starch-stearic acid mixture was stabilized by additional stirring for 30 min at 25 °C. Different concentration of stearic acid solutions in ethyl alcohol were also prepared (1%, 3%, 5%, and 7%), applied for ALC preparation at each temperatures (55, 65, and 75 °C), reacted for 60 min, and then used for the experiments.

### 2.3. Complexing Index (CI) of the Amylose-Lipid Complexes

The degree of ALC formation was determined by measuring the complexing index (CI) using an iodine solution, as described by Kaur and Singh [24]. The iodine solution used for analysis was prepared by dissolving 2 g of potassium iodide and 1.3 g I_2_ in 50 mL of distilled water and allowing them to dissolve overnight [25]. Then, distilled water was added to make up the volume to 100 mL. An amylose-lipid complex sample (5 g) was mixed with 25 mL distilled water in a test tube. The test tube was vortexed for 2 min and centrifuged for 20 min at 1500 × *g*. The supernatant (250 μL) and iodine solution (1 mL) were added to distilled water (7.5 mL) in a 15 mL test tube. The tube was vortexed, and the absorbance was measured at 690 nm. CI was calculated using the following equation:

(1) $$\mathrm{CI}\;\left(\%\right)=\frac{\left(\mathrm{Absorbance}\;\mathrm{of}\;\mathrm{control}-\mathrm{absorbance}\;\mathrm{of}\;\mathrm{sample}\right)}{\mathrm{Absorbance}\;\mathrm{of}\;\mathrm{control}}\times 100.$$

### 2.4. Separation of the Amylose-Lipid Complexes

After sample preparation, each sample was washed to remove free stearic acid and corn starch using the methods described by Chang, et al. [23] and Reddy, et al. [17] with some modification. ALC samples prepared as in Section 2.2 (250 g) were recovered by centrifugation (1500× *g*, 20 min) and successively washed with hot water and 50% ethanol (50 mL each) to remove free starch and free stearic acid. The final precipitates were dried at 40 °C overnight and milled using a cyclone mill.

### 2.5. X-Ray Diffraction (XRD)

The crystalline structure of the complexes was assessed using an X-ray diffractometer (D8 Advance, Bruker-AXS Co., Karlsruhe, Germany) via the method described by Seo, Kim, and Lim [19]. The scanning range and speed were 5–30° (2θ) in steps of 0.02° per second at a 40 kV target voltage and 40 mA current. All samples were measured after being dried and milled.

### 2.6. In Vitro Starch Digestibility

In vitro digestibility was determined using the Englyst procedure with modifications [26]. The dried samples (100 mg) were mixed with 1.75 mL water and boiled for 10 min. Sodium maleate buffer (3.5 mL, pH 6.0) was added, and the solution was equilibrated for 5 min at 37 °C; afterwards, 400 U pancreatin and 170 U amyloglucosidase were added. Aliquots were withdrawn at 20 and 120 min intervals and mixed with equal volumes of 95% ethanol. After centrifugation, the reducing sugar content in the supernatant was measured by the 3,5-dinitrosalicylic acid method. The ratios of rapidly digestible starch (RDS), slowly digestible starch (SDS), and resistant starch (RS) were determined based on their definition by Englyst, Englyst, Hudson, Cole and Cummings [26], which combines the G20 (glucose liberated after 20 min), G120 (glucose liberated after 120 min), FG (free glucose), and TG (total glucose) values, using the following formulas:

RDS (%) = (G20 − FG) × 0.9/TG SDS (%) = (G120 − G20) × 0.9/TG RS (%) = [TG − (RDS + SDS)]/TG.(2)

### 2.7. Preparing White Pan Bread with the Amylose-Lipid Complexes

Bread samples were prepared according to the AACC standard straight dough method with slight modifications [27]. The ingredients were as follows: 100 g of hard flour (Mildawon Co. Inc., Gongju, Korea), 6 g of refined sugar (CJ Co. Inc., Seoul, Korea), 6 g of shortening (Smart Baker Gold Shortening, Sinar Mas Co. Inc., Jakarta, Indonesia) for control bread, 1.5 g of sea salt, and 6 g of yeast. For test samples, 6 g of ALC or 3 g of shortening with 3 g of ALC were used for replacing shortening in bread formulation. The ingredients were first mixed for 3 min at low speed and then for 7 min at high speed (SK mixer, Tsubame, Japan). Subsequently, the dough was benched for 1 h at room temperature (25 °C). After sheeting, molding, and proofing for 50 min at 38 °C, the dough was baked at 175 °C for 35 min. All experiments were conducted in triplicate, and the loaves of bread were cooled until their internal temperature reached 30 °C before being used in the experiments.

### 2.8. Proximate Analysis and Physical Properties of Bread

Moisture content was determined using an infrared moisture analyzer (Infrared moisture determination balance FD-720, KETT Electric Laboratory, Tokyo, Japan). The ash, crude protein, and crude fat content of the ALC bread samples were determined using the methods recommended by AOAC International [28]. The calorie content was measured using an oxygen bomb calorimeter (Parr 6400 Automatic Isoperibol Calorimeter, Parr Instrument Inc., Co., Moline, IL, USA). The specific volume of bread was measured using a volscan profiler (Stable Micro Systems VolScan Profiler, Stable Micro systems Inc., Godalming, UK).

### 2.9. Differential Scanning Calorimetry (DSC)

The thermal properties of white pan bread were analyzed using a Diamond DSC (Perkin-Elmer, Inc., Waltham, MA, USA). The gelatinization parameters, including the onset temperature (To), peak temperature (Tp), conclusion temperature (Tc), and enthalpy change (ΔH), were calculated from the DSC thermograms. The ALC samples were measured after milling, and bread samples were ground into a powder after freeze-drying (FDU-1200, Eyela, Tokyo, Japan). The samples (7 mg) were weighed into the DSC sample pan (stainless steel pans, covers, and tygon O-rings; Perkin-Elmer, Inc., Waltham, MA, USA), and each pan was sealed after adding 21 μL distilled water. All sample pans were equilibrated at room temperature for 1 h and then heated from 10 to 150 °C at a rate of 10 °C/min.

### 2.10. Statistical Analysis

All experiments were repeated at least three times. Statistical analysis was performed using SPSS® statistics program ver. 25 (IBM, New York, NY, USA). Duncan’s multiple-range test in one-way analysis of variance (ANOVA) was carried out to identify significant differences (*p* < 0.05). Each value shown is the mean ± standard deviation (SD), and different letters indicate significant differences (*p* < 0.05).

## 3. Results

### 3.1. Complexing Index of the Starch-Lipid Complexes

The degree of starch-stearic acid complexing was assessed by calculating the complexing index (CI) using an iodine solution as a function of temperature, time, and ratio of stearic acid/starch. The effect of temperature and time was investigated using a 5% stearic acid to corn starch ratio at different temperatures (55, 65, and 75 °C) and durations of time (30, 60, and 120 min). The results are summarized in Table 1. At all temperatures, a 60 min reaction was the optimal complexation time for ALC preparation. The effects of varying the ratio of stearic acid/starch while maintaining the reaction time at 60 min are summarized in Table 2. In all trials, the CI values increased with higher ratios of stearic acid/starch; the values appeared to be saturated when the ratio was greater than 5%. Overall, a complexation reaction with 5% stearic acid/starch for 60 min at 75 °C was selected as the optimal condition for ALC preparation and used in the following experiments.

### 3.2. X-Ray Diffraction of the Amylose-Lipid Complexes

**Figure 1:**
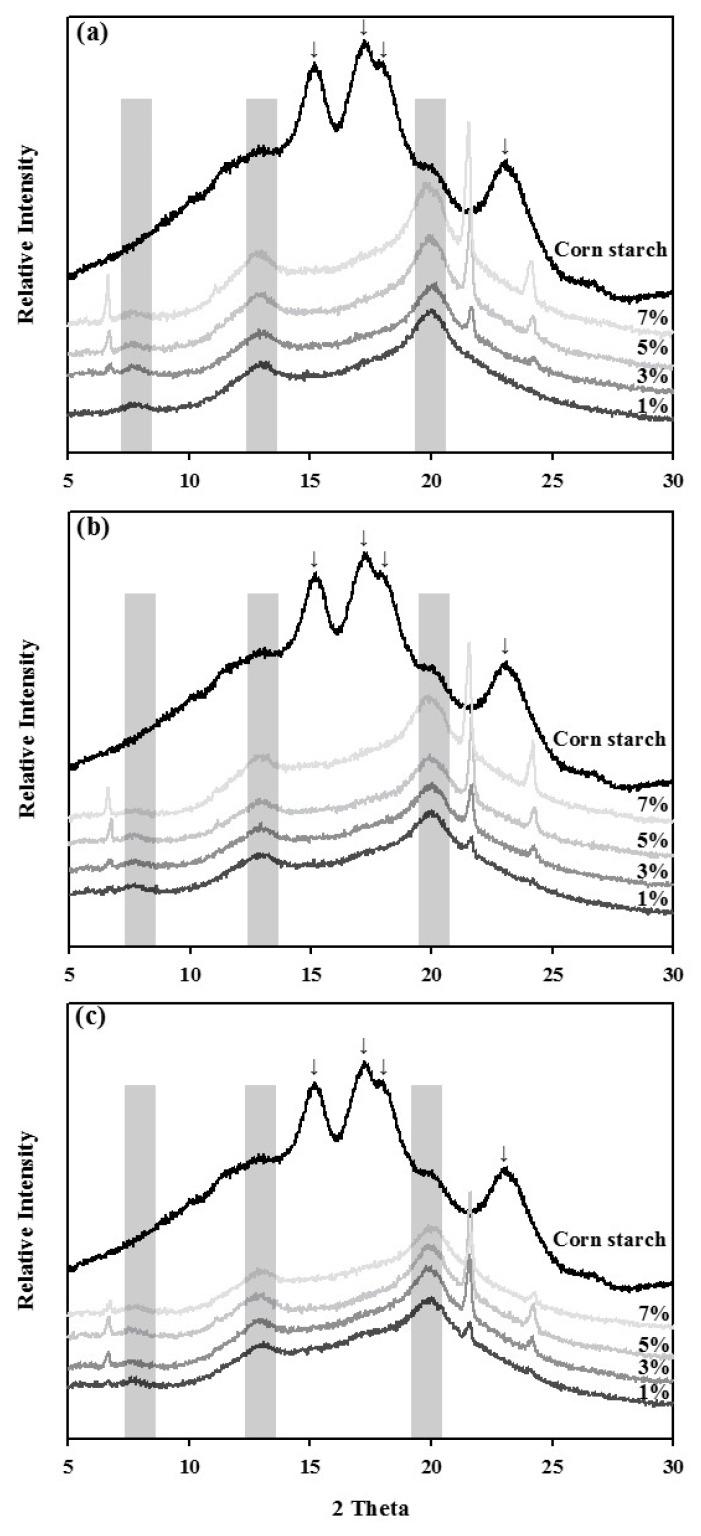
*X*-ray diffraction spectra of corn starch-stearic acid complexes prepared at different temperatures and with different stearic acid/corn starch ratios. (**a**) 75 °C, (**b**) 65 °C, (**c**) 55 °C.

The *X*-ray diffraction patterns of ALCs prepared under different conditions were compared to confirm the complexation of stearic acid into the starch structures (Figure 1). Characteristic peaks at Bragg angles (2θ) of 15.3, 17.2, 18.0, and 23.0° were observed with corn starch, which is an A-type starch; stearic acid complexation, however, shifted the XRD spectra. The ALC samples showed two main peaks at 13.0 and 20.0° and a minor peak at 7.0°, which is characteristic of a typical V-type complex [19]. Most of the major spectra in corn starch disappeared almost completely; thus, the new spectra generated by ALC formation indicated the existence of complexes. The peak intensities of the ALC samples increased with stearate concentration at all temperature ranges, with higher temperatures showing stronger intensities. The results were in the line with those of the CI value measurement.

### 3.3. Thermal Properties of the Amylose-Lipid Complexes

The degree of amylose-lipid complexation can also be confirmed via thermal analysis of the melting behavior of the ALCs. The thermal properties of the ALC samples treated at different temperature and concentrations were characterized using DSC (Table 3). The melting enthalpy (ΔH) of the ALC peak (90–108 °C) measures the amount of ALC formed during processing [29]. Enthalpies increased at higher stearate concentrations, which implied the enhancement of ALC formation. Of the tested temperatures, the highest level of ALC formation, like other attributes, such as CI values and XRD, occurred at 75 °C.

### 3.4. In Vitro Digestibility of the Amylose-Lipid Complexes

The in vitro digestibility of the ALC was determined using the enzymatic hydrolysis method; it was summarized as the relative portions of rapidly digestible starch (RDS), slowly digestible starch (SDS), and resistant starch (RS) after the in vitro digestion process (Table 4). Polydextrose was used as a resistant starch positive control for comparison. Normal corn starch contained 80% of RDS, 5.5% of SDS, and 0.3% of RS, whereas ALC had 76% of RDS, 7.1% SDS, and 1.3% of RS, indicating that the RS content in the ALC was 4.3 times higher than that in corn starch, which had a greater RDS and lower SDS.

### 3.5. Characteristics of Bread Prepared with the Amylose-Lipid Complexes

Proximate analysis results and calorie content of ALC-containing bread are summarized in Table 5. The crude protein and ash content were not affected by ALC replacement; however, the fat content was significantly reduced. The gross energy of the bread samples was also significantly reduced by ALC replacement: from 4515 to 4338 and 4186 cal/g with 50% and 100% replacement, respectively. The reduced calorie content of the ALC-containing bread was due to the reduced fat content after shortening replacement.

**Figure 2:**
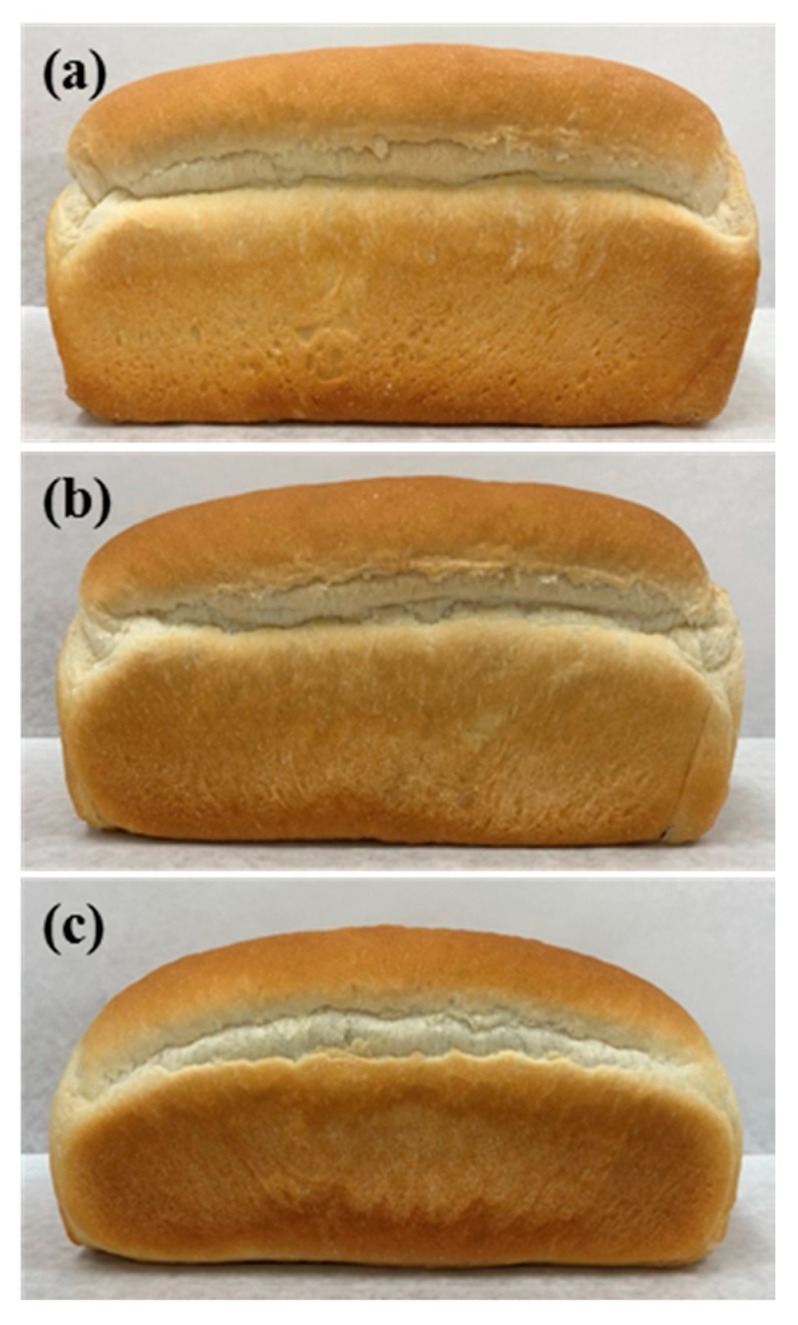
Overall shape and appearance of fresh loaves of amylose-lipid-containing breads. (**a**) Control bread; (**b**) ALC 50% bread was prepared with 50% of the shortening replaced with ALC; (**c**) ALC 100% bread was prepared with 100% of the shortening replaced with ALC.

The overall appearance and volume of these loaves were also compared with those of the control bread (Figure 2 and Table 5). Overall the shape of the loaves of bread was not significantly affected with 50% replacement of shortening (ALC 50% bread); however, it appeared to be altered by complete replacement (ALC 100% bread). Comparing the specific volume of the bread clearly revealed the effect of ALC on the overall appearance. In ALC 50% bread, the specific volume only decreased by 4%, whereas it was reduced by 23% in ALC 100% bread.

### 3.6. Starch Retrogradation in Bread during Storage

Quality changes in the ALC-containing bread during storage were assessed in terms of starch retrogradation (Table 6). Differential scanning calorimetry (DSC) was used to investigate the starch retrogradation in bread during storage (Table 6). The first peak (43–59 °C) indicated a melting endotherm of the crystal structure of retrograded starch, and the second peak (90–103 °C) represented an endothermic peak of amylose-lipid complex melting. Due to starch retrogradation, the enthalpy of peak 1 increased during storage in all samples, whereas ALC addition effectively retarded starch retrogradation after three days of storage. Replacement of shortening with ALC increased the content of the amylose-lipid complex, which was reflected in the enthalpy of peak 2 (as shown in Table 6). As the amount of added ALC increased, the enthalpy significantly increased with the increased amylose-lipid complex.

## 4. Discussion

Over the last several years, there has been an increase in the percentage of the population that is either overweight or obese. The growing concern about the relationships among health, feeding, and the maintenance of a healthy weight has boosted the market for foods with reduced energy values [30]. Amylose-lipid complex (ALC) is recognized as a promising replacement for low-fat food preparation [21,22,24,31,32]; however, to our knowledge, no attempt has been made to apply it to bread preparation. In this study, we prepared ALC with stearic acid and used it to replace shortening in white pan bread. Complexation with stearate was greater at higher temperatures, and it was found that the best condition for ALC preparation was a ratio of 5% of stearate to starch reacted for 60 min at 75 °C. Seo, Kim, and Lim [19] characterized ALCs with C18 fatty acids, including stearate; they reported that stearate was the most effective of the tested C18 fatty acids. Reaction temperature is an another critical factor in complex formation. Chang, He, and Huang [23] pointed out that complexation with a fatty acid and starch is significantly affected by the swelling power of the starch molecule, which will sharply increase from 60 °C to 75 °C.

The XRD spectra is a fingerprint for identifying the specific crystal structure of different types of starches. Corn starch, as an A-type starch, showed typical peaks at the Bragg angles (2θ) of 15.3, 17.2, 18.0, and 23.0° [33]. Complexation of starch with the fatty acid altered the XRD spectra as indicated in Figure 1, resulting in peaks at 7.0, 13.0, and 20.0°, which are a characteristic pattern of V-type starches [19]. Sufficient corn starch and stearic acid complexes were formed at all reaction conditions. Besides the typical peaks for ALC formation, additional peaks were also observed at 6.7, 21.5, and 24.2° in the ALC samples. These spectra might due to the presence of uncomplexed fatty acid in crystalline form at room temperature or pure stearate physically trapped in the starch molecule [34,35].

DSC was used to compare the forming ability of ALC under each condition. The melting enthalpy (ΔH) of the ALC peak appeared over 90 °C; the melting enthalpy (ΔH) of the amylose-lipid complex in corn starch was 0.77 J/g. For the ALC samples used in this study, the melting enthalpy of the ALC peak was significantly affected by temperature and the concentration of stearic acid. After ALC complexation, the melting enthalpies were 2.18–4.13 times greater that of normal corn starch. Higher melting enthalpies were observed with increasing temperatures and stearate concentrations, with the highest ΔH at 75 °C with 5% stearate; however, no significant difference was observed with over 3% stearate, which we assumed was the saturating starch/lipid concentration ratio.

ALC is recognized as resistant starch type 5 (RS5); Hasjim, et al. [36] summarized the preparation and application of this new type of RS5. They prepared RS5 with a high amylose debranched starch for efficient complexation and confirmed an increased RS content by ALC formation. In our study, the RDS content was decreased whereas SDS and RS content was significantly increased upon ALC formation. A similar report was made by Ai, et al. [37]. They investigated the in vitro digestion of octenyl succinic starch; the RS content of this starch increased and the RDS content reduced owing to complexation with normal corn starch. Increased levels of RS reduce the rate of digestion and absorption of carbohydrates and therefore lower the postprandial rise in blood glucose [21].

The overall shape and appearance of the ALC bread was not affected by 50% shortening replacement; however, 100% of shortening-replaced bread exhibited significant changes in whole shape and appearance. The specific volume of the ALC 100% bread was 23% lower than that of the control, but only a 4% reduction was observed in the ALC 50% bread. This reduction was due to the role of shortening in the structure development of bread. The solid fraction (fat crystals) in shortening is known to play an important role in the formation of the structure of the dough as it influences the volume, grain, and texture of the finished product [3]. In addition, shortening affects the increase in volume during baking; the increase in bread volume is reportedly difficult to achieve with most shortening substitutes [38]. Thus, the level of shortening replacement with ALC needs to be carefully considered.

The quality of bread is gradually reduced during storage and usually defined as “staling”, a term which indicates decreasing consumer acceptance of bakery products caused by changes in the crumb other than those resulting from the action of spoilage organisms [1]. The most widely adopted indicator of bread staling is the change in the crumb firmness by starch retrogradation, which is most frequently measured by thermal analysis. Melting endotherms (peak 1 in Table 6) can be used as a direct parameter of starch retrogradation, and ALC formation can also be determined with melting endotherms (peak 2 in Table 6) coming in at temperatures over 90 °C. Starch retrogradation progressed from day 2, and the increased ΔH of peak 1 was inversely correlated to the concentration of ALC, which implied that ALC formation affected starch retrogradation. Endothermic peak 2 originated from ALC formation was significantly increased by the addition of ALC, which inhibited starch molecule recrystallization, resulting in the retardation of starch retrogradation in bread. A similar report by Kweon, et al. [39] also mentioned that an increased amylose-lipid complex content can effectively retard starch retrogradation.

ALC addition at 50% replacement for shortening was expected to reduce fat content by 39% which can be clearly claimed as low-fat product, however, only a 4% reduction of calories was observed in ALC 50% bread. Further studies are required to optimize the formulation generating highly reduced calorie bakery product based on ALC replacement. Shortening replacement with other substitutes revealed that a certain level of each substitute can be applied in bakery products. Oatrim was used in cake preparation as a shortening replacement, and 20% of replacement was indicated as the maximum level without quality change [9]. Kim et al. [10] attempted to replace shortening with oleogels and concluded that 25% of was the limit of substitution without quality loss. In this study, we confirmed that 50% replacement of shortening with ALC is possible without quality change in white pan bread, which will expand the further applications of ALC in bakery products.

## 5. Conclusions

This study aimed to reduce the amount of shortening in white pan bread for industrial application by replacing it with amylose-lipid complexes without quality changes. The reaction time, temperature, and starch/lipid ratio of the corn starch and stearic acid complexes were examined to establish the optimum conditions. The corn starch and stearic acid complexing reaction at 75 °C for 60 min showed the highest complexing index of the tested conditions. The physical properties, proximate composition, and starch retrogradation during storage of ALC-containing breads were compared with those of the control bread. Crude fat and calories were decreased in the ALC-containing bread. However, the specific volume of the ALC 100% bread was significantly different from that of the control bread. The ALC 50% bread showed a reduced calorie content without volume changes, with a retardation in the starch retrogradation during storage. Collectively, the results indicate that 50% replacement of shortening with ALC has positive effects on white pan bread and could be used for the preparation of low calorie, low-fat bakery products without quality changes. Therefore, ALC was shown to be a promising shortening replacement in bread preparation.

## Figures and Tables

**Table 1:** Effect of time and temperature on amylose-lipid complex formation with stearic acid and corn starch.

Conditions			Complexing Index (%)
55 °C	5% *	30 min	67.86 ± 0.73 ^a^
		60 min	71.21 ± 0.08 ^bc^
		120 min	68.87 ± 3.75 ^ab^
65 °C	5% *	30 min	69.25 ± 0.57 ^ab^
		60 min	75.53 ± 0.25 ^d^
		120 min	72.48 ± 0.43 ^c^
75 °C	5% *	30 min	77.64 ± 1.81 ^d^
		60 min	84.95 ± 0.06 ^f^
		120 min	81.55 ± 1.46 ^e^

* 5% of stearic acid/corn starch was used for complexation. Means with different superscripts (a–f) in the same column were significantly different (*p* < 0.05).

**Table 2:** Effect of the stearic acid/corn starch ratio on amylose-lipid complex formation.

Conditions			Complexing Index (%)
55 °C	1% *	60 min	22.90 ± 1.66 ^a^
	3%	60 min	65.66 ± 0.65 ^de^
	5%	60 min	71.21 ± 0.08 ^f^
	7%	60 min	66.13 ± 0.31 ^de^
65 °C	1%	60 min	31.62 ± 1.99 ^c^
	3%	60 min	64.69 ± 0.83 ^d^
	5%	60 min	75.53 ± 0.24 ^h^
	7%	60 min	67.46 ± 0.36 ^e^
75 °C	1%	60 min	27.96 ± 2.01 ^b^
	3%	60 min	73.82 ± 0.63 ^gh^
	5%	60 min	84.95 ± 0.06 ^i^
	7%	60 min	73.44 ± 0.39 ^g^

* Different ratio of stearic acid/corn starch was used for complexation. Means with different superscripts (a–i) in the same column were significantly different (*p* < 0.05).

**Table 3:** Thermal properties of amylose-lipid complexes (ALCs) prepared under different conditions.

Conditions		ALC Peak			
		To (°C)	Tp (°C)	Tc (°C)	ΔH (J/g)
55 °C	1%	92.98 ± 0.41	100.88 ± 0.28	106.33 ± 1.45	1.68 ± 0.73 ^a^
	3%	95.06 ± 3.99	102.73 ± 1.17	107.76 ± 0.14	2.63 ± 0.30 ^ab^
	5%	91.30 ± 2.37	101.64 ± 0.58	107.70 ± 0.93	2.24 ± 0.75 ^ab^
	7%	94.63 ± 1.31	101.92 ± 0.88	107.63 ± 0.68	2.21 ± 0.50 ^ab^
65 °C	1%	90.84 ± 3.09	100.80 ± 0.33	106.29 ± 0.76	1.72 ± 0.04 ^a^
	3%	92.02 ± 1.58	101.40 ± 0.10	107.22 ± 0.44	2.21 ± 0.28 ^ab^
	5%	92.76 ± 2.25	101.61 ± 0.52	107.16 ± 0.78	2.15 ± 0.39 ^ab^
	7%	94.11 ± 0.76	102.07 ± 0.81	107.93 ± 0.87	2.14 ± 0.75 ^ab^
75 °C	1%	93.32 ± 1.58	101.57 ± 0.41	106.98 ± 0.98	2.37 ± 0.94 ^ab^
	3%	92.17 ± 1.51	102.12 ± 0.41	107.96 ± 0.23	3.18 ± 0.51 ^b^
	5%	93.76 ± 0.92	101.95 ± 0.59	107.63 ± 0.13	3.97 ± 0.12 ^b^
	7%	93.79 ± 0.54	102.58 ± 0.43	108.29 ± 0.57	2.90 ± 0.26 ^b^

ALCs were prepared with different ratio of stearic acid/corn starch for 60 min at different temperatures. Means with different superscripts (a–g) in the same column were significantly different (*p* < 0.05).

**Table 4:** Percentages of RDS, SDS, and RS in corn starch and the amylose-lipid complex.

	Corn Starch	Amylose-Lipid Complex(ALC)	Polydextrose
RDS (%)	80.2 ± 0.61 ^c^	76.6 ± 1.66 ^b^	4.30 ± 0.41 ^a^
SDS (%)	5.50 ± 0.38 ^a^	7.10 ± 1.98 ^a^	0.50 ± 0.39 ^b^
RS (%)	0.30 ± 0.23 ^a^	1.30 ± 0.09 ^b^	94.60 ± 0.25 ^c^

RDS, rapidly digestible starch; SDS slowly digestible starch; RS, resistant starch. ALC sample was prepared with 5% of stearic acid/corn starch for 60 min at 75 °C. Means with different superscripts (a–c) in the same row were significantly different (*p* < 0.05).

**Table 5:** Approximate composition of amylose-lipid complex-containing bread.

BreadSamples	CrudeProtein (%)	Crude Fat (%)	Ash (%)	Calorie (cal/g)	Specific Volume (mL/g)
Control	9.77 ± 0.04 ^a^	4.99 ± 0.21 ^b^	1.06 ± 0.05 ^a^	4515.20 ± 5.50 ^c^	6.66 ± 0.11 ^c^
ALC 50%	9.80 ± 0.03 ^a^	3.04 ± 0.21 ^ab^	1.08 ± 0.07 ^a^	4338.00 ± 12.21 ^b^	6.39 ± 0.09 ^b^
ALC 100%	9.65 ± 0.13 ^a^	1.28 ± 0.18 ^a^	1.03 ± 0.07 ^a^	4186.25 ± 4.58 ^a^	5.16 ± 0.03 ^a^

ALC 50% indicate a bread prepared with 50% of shortening replacement with ALC. ALC 100% indicate a bread prepared with 100% of shortening replacement with ALC. Means with different superscripts (a–c) in the same column were significantly different (*p* < 0.05).

**Table 6:** Thermal properties of amylose-lipid-containing bread during storage at room temperature.

		Peak 1				Peak 2			
		To (°C)	Tp (°C)	Tc (°C)	ΔH (J/g)	To (°C)	Tp (°C)	Tc (°C)	ΔH (J/g)
**Day 2**	**Control**	45.4 ± 0.38	53.8 ± 1.13	59.5 ± 0.41	1.1 ± 0.12 ^a^	92.8 ± 1.19	98.1 ± 0.63	102.9 ± 0.29	0.3 ± 0.03 ^a^
	**ALC 50%**	44.0 ± 2.24	53.9 ± 0.87	59.6 ± 0.13	1.2 ± 0.10 ^a^	91.0 ± 0.68	97.6 ± 0.39	103.2 ± 0.68	0.6 ± 0.08 ^b^
	**ALC 100%**	48.1 ± 1.68	54.5 ± 0.27	59.4 ± 0.71	1.0 ± 0.28 ^a^	91.8 ± 0.34	98.7 ± 0.34	103.5 ± 0.12	0.9 ± 0.06 ^c^
**Day 3**	**Control**	45.0 ± 0.81	53.9 ± 0.49	59.7 ± 0.46	1.7 ± 0.17 ^a^	91.7 ± 0.40	97.8 ± 1.09	103.3 ± 0.56	0.4 ± 0.08 ^a^
	**ALC 50%**	45.4 ± 0.23	53.5 ± 0.63	59.5 ± 0.11	1.6 ± 0.19 ^a^	91.7 ± 1.81	98.1 ± 0.74	103.1 ± 0.29	0.6 ± 0.06 ^b^
	**ALC 100%**	45.7 ± 0.80	54.2 ± 0.30	59.8 ± 0.61	1.5 ± 0.22 ^a^	92.4 ± 0.44	99.0 ± 0.10	103.6 ± 0.09	0.9 ± 0.08 ^c^
**Day4**	**Control**	45.3 ± 0.27	53.7 ± 0.39	59.4 ± 0.24	1.9 ± 0.05 ^b^	93.2 ± 2.63	97.7 ± 0.43	103.3 ± 0.24	0.4 ± 0.01 ^a^
	**ALC 50%**	45.1 ± 0.03	54.1 ± 0.19	59.6 ± 0.42	1.7 ± 0.06 ^a^	91.9 ± 0.96	97.7 ± 2.33	103.4 ± 0.33	0.6 ± 0.06 ^b^
	**ALC 100%**	45.5 ± 0.65	53.9 ± 0.59	59.8 ± 0.06	1.7 ± 0.10 ^a^	92.2 ± 0.42	98.6 ± 0.51	103.4 ± 0.16	0.9 ± 0.03 ^c^

Control indicate normal white pan bread. ALC 50% indicate a bread prepared with 50% of shortening replacement with ALC. ALC 100% indicate a bread prepared with 100% of shortening replacement with ALC. Means with different superscripts (a–c) in the same column indicate difference significantly between ALC replacement ratio in the same day (*p* < 0.05).
